# Assessment of Referrals into the Soft Tissue Sarcoma Service: Evaluation of Imaging Early in the Pathway Process

**DOI:** 10.1155/2012/781723

**Published:** 2012-06-26

**Authors:** Emma Rowbotham, Shaheel Bhuva, Harun Gupta, Philip Robinson

**Affiliations:** ^1^Department of Radiology, Leeds Teaching Hospitals, Leeds LS1 3EX, UK; ^2^Musculoskeletal Centre, X-Ray Department, Chapel Allerton Hospital, Leeds Teaching Hospitals, Leeds LS7 4SA, UK

## Abstract

*Purpose*. To prospectively evaluate regional referrals into a soft tissue sarcoma service from outside the tertiary centre with local hospital imaging. *Materials and Methods*. Consecutive referrals were prospectively assessed for: patient demographics, source, referral date, date received by Multidisciplinary Team (MDT), lesion size, local radiology, MDT radiology and final diagnoses. Radiology diagnosis was categorised benign, indeterminate or malignant by consensus. Delays were defined as >10 days. *Results*. 112 patients were included with high correlation between local and MDT radiology categrorisation and histology (*P* = 0.54 and *P* = 0.49, resp.). There was only a trend for MDT radiology diagnosis to downgrade local imaging diagnosis (*n* = 15, *P* > 0.05). 48 cases (43%) had ultrasound and MRI at referral and 20 (18%) ultrasound only. 85% of cases were benign (lipoma most common), 15% malignant (sarcoma most common). Delay occurred in 34% of cases. *Discussion*. In comparison to previous series these results show a reduction in benign lesions, increased biopsy and malignancy rate for lesions referred to a tertiary centre when imaging is performed and reviewed by local radiologists. *Advances in Knowledge*. Imaging triage of soft tissue masses can decrease benign referral rates and increase the proportion of indeterminate and malignant lesions referred to specialist centres.

## 1. Introduction

Soft tissue sarcomas account for approximately 1% of all adult primary tumours, which equates to approximately 2000 cases per year in the UK [1, 2]. In the majority of cases there is no known aetiology, although rarely there is a recognised link with various genetically linked disorders (e.g., neurofibromatosis, tuberous sclerosis) [3] and previous radiotherapy. 

Management of soft tissue sarcomas is focussed in tertiary centres, where at least 100 new sarcomas are treated per year. A far greater number of patients than this present to primary and secondary care with clinically suspicious soft tissue masses, which are ultimately not sarcomas, but require further clinical and radiological investigation.

Previous studies have suggested unacceptable delays in presentation and clinical guidelines have been published stating which soft tissue mass characteristics are concerning (Table 1) and prompt urgent referral to a tertiary centre [2, 4]. Current Department of Health guidelines also suggest that soft tissue lesions satisfying these criteria should be referred under the “2 week” rule [4].

In our region, a soft tissue sarcoma pathway was established in 2010 for both GP's and secondary care clinicians and states all relevant imaging investigations should be performed locally prior to tertiary referral.

The aim of this study was to prospectively evaluate all regional referrals from outside the tertiary centre hospital with local hospital imaging looking particularly at the source of referral, referral timings, the number of biopsies performed, the rate of benign versus malignant cases, and the level of agreement between the provisional imaging diagnosis (referrer and Multidisciplinary Team (MDT)) and the final diagnosis.

## 2. Methods

Consecutive referrals, over a six-month period, into a regional soft tissue sarcoma service from outside the tertiary hospital were evaluated. Institutional ethics committee approval stated that individual patient consent was not necessary.

The following data was collected for each case: patient demographics, source and date of referral, date of referral receipt by MDT staff, details of imaging performed prior to referral, provisional radiology diagnosis, MDT date, radiology MDT, and final diagnoses.

### 2.1. Pathway Timings

The timing between last local imaging performed and the referral being made were calculated as well as the time between source referral date and date received by MDT staff. Delays at either of these stages were categorised into <10 days (no delay) or >10 days (delay).

### 2.2. Imaging Assessment and Correlation

The initial local radiology reports were assessed for provisional radiological diagnosis and categorised as benign, indeterminate, or malignant.

Imaging studies were also evaluated by 2 MSK radiologists (12 and 3 years of experience), blinded to the provisional diagnosis and categorised as benign, indeterminate, or malignant by consensus using established criteria (Table 2) [5, 6]. Lesions in categories 1–5 were considered benign, categories 6 and 7 as indeterminate, and those in category 8 as malignant.

### 2.3. Correlation

Core biopsy, open biopsy, and/or resection histology reports were documented following the procedure. Correlation between the local radiology diagnosis, MDT radiology diagnosis, and final diagnoses was analysed using the Chi-square test. This was performed using the pathology results as the expected finding and comparing both the provisional and MDT diagnoses with this, respectively (95% confidence limits). The patients were categorised according to final diagnosis to see if the provisional diagnosis had an influence on the urgency/speed of referral.

## 3. Results

112 (61 males, 51 females, mean age 52 years) patients were referred from outside the main tertiary centre in the 6-month study period. 40/112 (36%) of cases were referred from primary care and 72/112 (64%) were referred by a hospital clinician.

### 3.1. Pathway Timings

The length of time between imaging and the subsequent referral to the MDT ranged from 1 to 180 days with a mean of 11.7 days and a mode of 1 day. A small number of cases were delayed for >100 days hence the significant difference seen between the mean and the mode. No delay was seen in 67% of the referred patients. There was no established pattern in terms of which patients experienced a delay in the referral pathway.

### 3.2. Imaging Assessment and Correlation

All cases had either ultrasound or MRI performed prior to referral. 48 cases (43%) had both ultrasound and MRI performed prior to referral whilst 20 (18%) had only ultrasound; of these cases it was felt ultrasound was sufficient in 16 cases however in the remaining 4 cases MRI was performed following initial MDT discussion of the case. In each of these cases MRI was required to more accurately assess the depth of the lesion or its anatomical relations to other structures but did not alter the MDT radiology diagnosis as determined on ultrasound imaging. 44 (39%) had only MRI performed and none of these patients were referred for additional ultrasound imaging following MDT discussion.

### 3.3. Benign versus Malignant Rate

**Figure 1:**
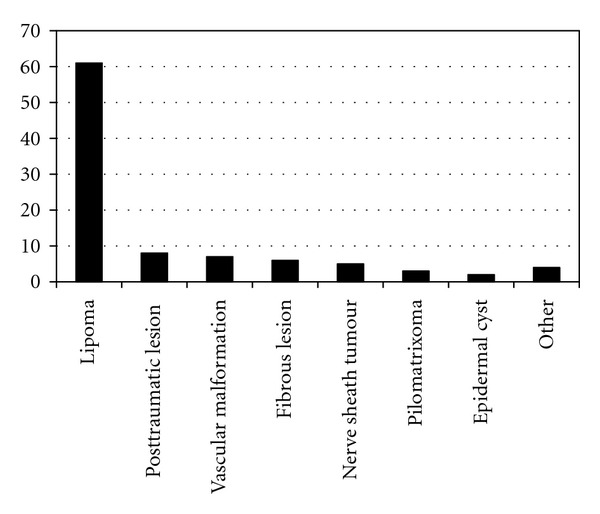
Graph to show the distribution of benign cases.

**Figure 2:**
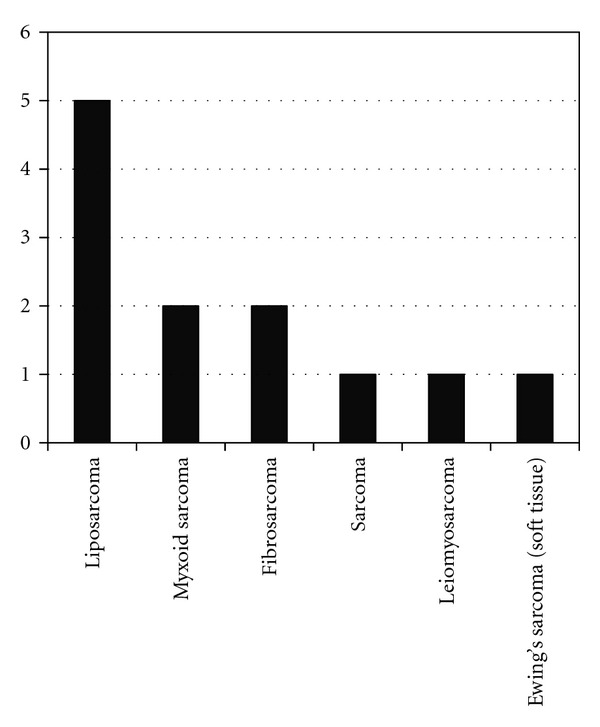
Graph to show the distribution of sarcoma subtypes.

**Figure 3:**
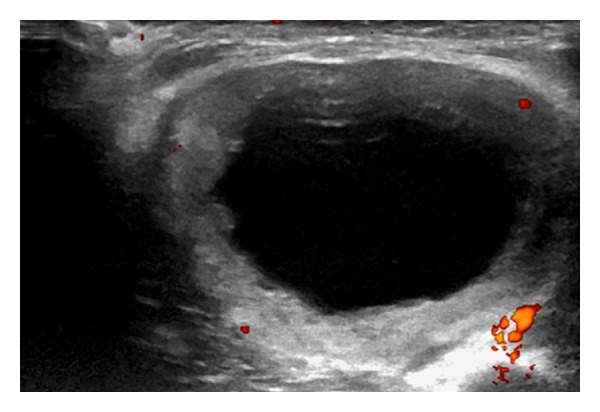
Transverse ultrasound image of the upper arm in a 65-year-old lady presenting with a soft tissue mass. A hypoechoic central portion is seen surrounded by a solid rim with internal vascularity. The lesion was indeterminate on imaging but was found to be a squamous cell carcinoma metastasis on histology.

Of the 112 patients studied the majority of cases had a benign final diagnosis 95/112 (85%) with lipoma being the most common diagnosis in this group (Figure 1). 49/112 (44%) patients underwent biopsy of their soft tissue lesion and of these 17/49 (35%) had a malignant diagnosis; sarcoma (*n* = 13) (Figure 2), soft tissue metastases (*n* = 4) (breast cancer, squamous cell carcinoma, (Figure 3) and colonic adenocarcinoma).

Histology was available for 92 of the 112 patients in the study. In 20 patients no pathology was available; in these cases the patient was either referred back to the base hospital for conservative management or removal of a benign lesion or a decision was made to perform serial imaging followup as opposed to biopsy or excision. On clinical and radiological followup after 12 months none of these masses had changed significantly and no sarcomas were excised elsewhere in the region in this patient group.

Comparing the local referrer provisional categorisation diagnosis with histopathology and MDT imaging diagnosis with histopathology showed no significant difference between diagnosis by either the referrer or the MDT radiologists compared with final histology (*P* = 0.54 and *P* = 0.49, resp.). These calculations are based on the assumption that pathology for the 20 cases which were not excised would follow the same distribution as the cases for which histology was available. Although there was no statistical difference in referrer and MDT imaging diagnoses and final pathology there was a trend for the MDT imaging diagnosis to downgrade the referrer imaging diagnosis to benign. 11 of these cases were downgraded to benign lipoma (see below); further 4 cases were downgraded to benign at MDT and included 2 inflammatory masses, nodular fasciitis, and myositis ossificans.

### 3.4. Lipomas

**Figure 4:**
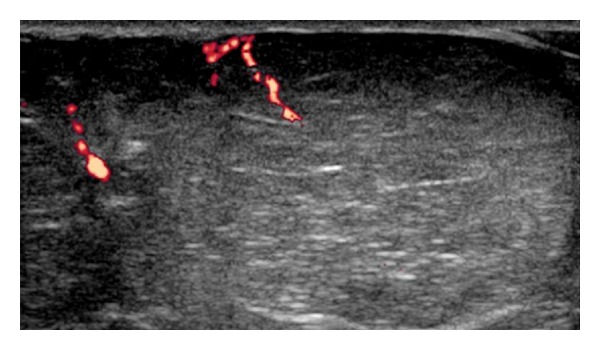
Ultrasound image of a superficial encapsulated lipoma with vascularity seen along the internal septae. The local reporting radiologist referred this lesion due to the internal vascularity. MDT determined the lesion benign on the basis of imaging and clinical information.

Referral criteria for the 61 lipomas in this study are presented in Table 3 with the majority 34/61 (56%) referred due to their size only (>5 cm). 11 cases were referred as a result of concerning features on local imaging; 5 cases due to multiple internal septae (Figure 4), 2 cases due to prominent internal vascularity, 1 case with both internal septae and internal vascularity, 1 case with heterogenous echotexture, and 2 cases which showed enhancement following intravenous Gadolinium contrast administration. The MDT radiology categorization diagnosis determined all these lesions to be benign. 

## 4. Discussion

Development of regional soft tissue sarcomas centres with focussed expertise are deemed necessary in the UK due to the rare nature of these tumours. NICE guidance for improving outcome has focused primarily on guidelines and care pathways following diagnosis [2]. There remains no nationally agreed structure for the initial investigation and management of soft tissue masses which present to primary care with the vast majority of referrals to regional sarcoma services being benign lesions. At our institution a total of 1708 cases were discussed at the Sarcoma MDT in the last 12-month period of which 109 (6%) were newly diagnosed sarcomas. A study carried out in 2008 suggested that ultrasound was an effective triage tool in the investigation of soft tissue lesions [6], and when used in conjunction with MRI most soft tissue lesions referred from primary care can be characterised as either benign, indeterminate, or malignant by local radiologists. In comparison to that series the current study showed a reduction in benign lesions (85% versus 95%), a reduction in normal/cystic lesions (5% versus 34%), increase in biopsy rate (44% versus 8%), and increase in malignant lesions (15% versus 2%) with imaging reviewed by local radiologists and then referred to the tertiary centre. These figures also reflect the fact that guidelines issued following this study [6] were agreed with radiologists in the referring centres before a local pathway was instituted and have been implemented effectively. In addition the result of this guidance could have resulted in the lack of statistical difference between the provisional categorization diagnoses provided by the referring radiologist's report and the MDT radiologist's diagnosis.

The majority of the radiology literature regarding soft tissue sarcoma characterization has focussed on MR imaging. Previous studies have shown that MR imaging can provide a high degree of sensitivity and specificity in characterising soft tissue tumours as either benign or malignant (82 and 93%, resp.) [7, 8]. Correlation of the provisional diagnosis with the final diagnosis in this study was high, whether by US or MRI, confirming the importance of completing imaging prior to referral ensuring both appropriate referral and prioritisation for discussion. Only four cases referred with ultrasound imaging only subsequently underwent MRI following MDT discussion to more accurately delineate the lesions' anatomical position prior to surgical excision. This may indicate that if both the diagnosis and anatomical relations of a lesion can be confidently reported on ultrasound alone then there is no routine need for MRI. A larger cohort study would be needed in order to assess accuracy of both these parameters on ultrasound alone however in most centres this would offer a more flexible and rapid service than MRI.

Lipomas are primarily referred into specialist MDT as a result of NICE guidance (Table 1). Although this study showed a reduction in comparison to previous series there was still a large number of ultimately benign lipomas referred, mainly, due to their size. Evidence for referral of lesions over 5 cm is documented in various studies which have looked at risk factors for malignant lesions. One study showed 10% of malignant lesions in the study were <5 cm and 65% benign lesions were >5 cm showing this is not a completely discriminatory criterion [9]. In our small sample all lipomas were ultimately benign if either MRI or ultrasound showed no concerning imaging features. Further work will be necessary in this area but this would suggest that lipomatous lesions meeting referral criteria on size alone and no further radiological or clinical concerning features could be managed locally, if surgical expertise allowed. This could significantly reduce the number of benign cases being referred for management and treatment at tertiary centres.

It is recognised that a delay in presentation or referral and subsequent diagnosis is a significant cause of morbidity for sarcomas [10]. However, it is still difficult to quantify the delay duration which becomes significant in terms of sarcoma outcome. A recent study quantifying delays between initial symptoms and specialist referral showed that 73% of patients present to a GP within 3 months of symptom onset and 50% of patients are referred for specialist opinion within 1 month of presentation [11]. Another study suggested that all sarcoma patients treated were having their first investigation within 62 days of referral regardless of whether a 2-week wait or routine referral was used [12]. Initial local imaging of patients aspires to the DOH Cancer Group vision of 2012 [13] and allows patients immediate reassurance if benign with prioritisation of the cases which are not. Without imaging clinical examination alone could result in unnecessary patient concern and travel to specialist centres further reducing their ability to efficiently deal with indeterminate or suspicious lesions. Although not directly evaluated by this study, delays could potentially be further reduced if all imaging was commissioned by primary care without secondary care referral. Benign lesions could be managed locally, in local secondary care if necessary, and other lesions referred to the sarcoma centre.

Limitations of this study include lack of detail on patient delay in presenting to primary care and patients with local normal or nontumour imaging findings that were never referred onwards. However there has been only one other sarcoma pathology recorded in the region, outside the tertiary hospital pathway, and that patient had no preoperative imaging. Pathology was not available in all cases but this reflects the prospective nature of this study and the offer to local radiologists to have a low threshold for referring indeterminate lesions. However, this is a potential bias in previous studies which retrospectively evaluate imaging of lesions via pathology databases.

Although possible algorithms have been suggested for the investigation of soft tissue masses [14], defining an efficient referral pathway for all patients within a large region is challenging. The results of this study suggest that early imaging within a designated pathway should help prioritise onward referral for suspicious lesions and help reduce the volume of benign lesions referred.

## Figures and Tables

**Table 1:** Clinical criteria indicating that further investigation of a soft tissue mass is required [2].

Pain attributable to lesion
Mass > 5 cm
Mass deep to deep fascia
Mass increasing in size

**Table 2:** Ultrasound/MRI diagnostic categories 1–8.

Category name description
(1) Normal—no abnormality seen.
(2) Benign cyst or ganglion cyst.
(3) Benign vascular lesion.
(4) Benign other—any lesion with either inflammatory characteristics or benign soft tissue mass.
(5) Lipoma—homogeneous lesion, none or linear septal vascularity, and no concerning clinical features.
(6) Lipoma requiring further evaluation:
(i) clinically painful, enlarging, >5 cm in size, deep, and/or
(ii) lipoma, but mild heterogenicity on ultrasound or MRI.
(7) Indeterminate—clinically painful, deep, >5 cm and/or enlarging solid mass, no Doppler flow.
(8) Possible sarcoma—solid, heterogeneous lesion, distortion of surrounding anatomy, and disorganized power Doppler flow.

**Table 3:** Table to show the number of lipoma referrals due to size, depth, concerning imaging features and symptoms.

Size	34 (57%)
Depth	12 (20%)
Imaging features	11 (18%)
Symptomatic	4 (6%)
